# A virus-induced gene silencing (VIGS) system for functional genomics in the parasitic plant *Striga hermonthica*

**DOI:** 10.1186/1746-4811-10-16

**Published:** 2014-06-03

**Authors:** Dinah Kirigia, Steven Runo, Amos Alakonya

**Affiliations:** 1 Jomo Kenyatta University of Agriculture and Technology, Institute of Biotechnology Research, P. O. Box 62000-00200, Nairobi, Kenya; 2 Department of Biochemistry and Biotechnology, Kenyatta University, P.O. Box 43844, 00100 Nairobi Kenya

**Keywords:** *Striga hermonthica*, Viral induced gene silencing, Agro-drench, Agro-infiltration, Tobacco rattle virus, *Phytoene desaturase*

## Abstract

**Background:**

*Striga hermonthica* is a hemiparasitic weed that infects cereals in Sub Sahara Africa (SSA) resulting in up to 100% grain yield loss. This significant loss in grain yields is a major contributor to food insecurity and poverty in the region. Current strategies to control the parasite are costly, unavailable and remain unpracticed by small-scale farmers, underscoring the need for more economical and sustainable control strategies. Development of resistant germplasm is the most sustainable strategy in the control of *S. hermonthica*, but is constrained by paucity of resistance genes for introduction into crop germplasm. RNA interference (RNAi) has potential for developing host-derived resistance against *S. hermonthica* by transformation of host crops with RNAi sequences targeted at critical Striga genes. The application of RNAi in management of *S. hermonthica* is however constrained by lack of efficient high throughput screening protocols for the candidate genes for silencing, as well as sub optimal delivery of siRNAs into the parasite. In comparison to stable transformation, viral induced gene silencing (VIGS) is a rapid and powerful tool for plant functional genomics and provides an easy and effective strategy in screening for putative candidate genes to target through RNAi. In addition, VIGS allows for a secondary amplification of the RNAi signal increasing the siRNA threshold and facilitates siRNA transport through viral movement proteins. We tested the efficiency of the Tobacco *rattle virus* (TRV1 and TRV2) VIGS vectors in silencing *S. hermonthica phytoene desaturase* (PDS) gene through agrodrench and agro-infiltration.

**Results:**

We report the validation of VIGS in *S. hermonthica* using a silencing cassette generated from *TRV* with a *PDS* gene insert. Agro-infiltrated and agro-drenched *S. hermonthica* leaves showed photo-bleaching phenotypes typical for PDS silencing within 7 and 14 days post infection respectively. In both cases *S. hermonthica* plants recovered from photo-bleaching effects within 28 days post inoculation. The transformation efficiency of the VIGS protocol in *S. hermonthica* was (60 ± 2.9)%.

**Conclusion:**

These results demonstrate that the TRV-VIGS system work in *S. hermonthica* and can be used for candidate gene validation for their role in the parasite development and parasitism, with the ultimate goal of developing resistant transgenic maize.

## Background information

Maize is an important staple food crop for majority of people in Sub-Sahara Africa (SSA) [1,2]. Maize grain yields are below the demand hence leading to food insecurity and poverty in the region. The low maize yields result from various biotic and abiotic factors that combined cause cereal grain loss worth of US$3 billion annual [3-5]. The most devastating biotic constraint to maize production in SSA is *Striga hermonthica* (Del.) Bentha, a root hemi-parasitic weed of maize which causes up to 100% grain loss annually [3,6,7]. The life cycle of *S. hermonthica* is intimately synchronized with that of its host, and the seeds of the parasite only germinate in response to chemical signals present in root exudates of the host [8]*. Striga hermonthica* infects maize by forming haustoria connections with the host vasculature resulting in syphoning of water and nutrients [8,9]. Although some *S. hermonthica* control strategies have been proposed and practiced, Striga seed bank in soils has continued to build up and the parasite has continued to spread to previously non-infected arable land [10,11].

Genetic engineering through cross species RNA interference (RNAi) technology offers great promise in parasitic plant management [12-19]. However, its applicability in *S. hermonthica* management has been constrained by lack of methods to deliver the silencing molecules and the lack of candidate genes to target [20]. The recent report on horizontal gene transfer from *Sorghum bicolor* to *S. hermonthica* has increased prospects of delivering the silencing RNA molecules from cereal hosts to *S. hermonthica*[21]. This suggests the possibility of RNAi in some monocots which have been reported to be recalcitrant to transformation. Host-derived resistance using RNAi dependent on efficient delivery of siRNAs from the host to the parasite in order to determine if the gene causes an alteration in the parasites’ phenotype, reviewed in [15]. An alternative approach to determine if a gene has a function on the parasite would be to develop a high throughput genetic transformation protocol for Striga. These two approaches present challenges, as transformation of grasses (especially rice, maize wheat and sorghum) is recalcitrant [22-26] and no protocols exist for Striga transformation yet.

Viral induced gene silencing (VIGS) is a technique that employs recombinant viruses to specifically reduce endogenous gene activity through plant innate silencing mechanisms called Post-Transcriptional Gene Silencing (PTGS) [27]. The VIGS vectors are usually standard binary Ti-plasmids that contain a viral genome and a fragment of the host plant’s target gene. The vectors are introduced in the plants via *Agrobacterium tumefaciens* infection that results in the transfer of the T-DNA containing the viral genome into the host genome of at least one cell, where it is transcribed, and translated [28,29] . This leads to the production of double-stranded RNAs (dsRNAs) due to self-assembly of viral ssRNA into hairpins or complementary sequences derived from sense and antisense viral ssRNA strands [30]. Dicer-like proteins cleave these viral dsRNAs into short interfering RNAs (siRNA) duplexes of 21–24 nucleotides (nt) in length [27-29]. These siRNAs are incorporated into a RNA-induced Silencing Complex (RISC) that guide and cleave complementary RNAs [31,32]. The virus-derived silencing signal is amplified and spreads systemically throughout the plant [33]. Amplification of VIGS results in down-regulation of target gene [27,34]. VIGS is not a stable transformation strategy but works transiently and therefore could be used as a powerful and rapid tool in gene validation for loss-of-function.

We report efficient Tobacco *rattle virus* (TRV-(1&2) VIGS vectors in silencing *S. hermonthica Phytoene desaturase (PDS)* gene. These findings have far reaching application in designing RNAi strategies based on host derived resistance.

## Results

### VIGS induced RNAi on *S. hermonthica* PDS causes photo-bleaching

**Figure 1:**
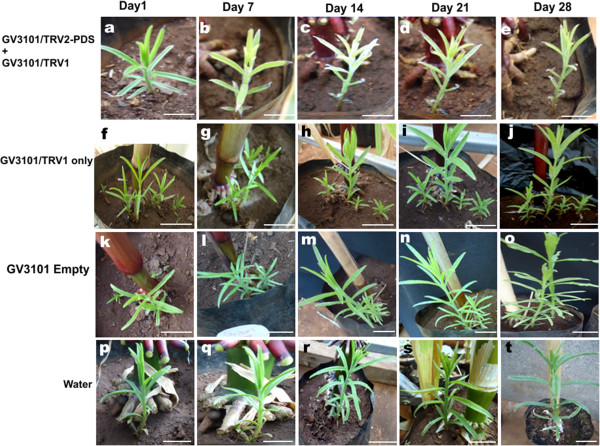
**Viral induced gene silencing (VIGS) via Agro-drench method on *S. hermonthica*. a**, **b**, **c**, **d**, and **e** are plants agro-drenched with *Agrobacterium* strain GV3101 with TRV2- PDS (GV3101/TRV2-PDS), and mixed in 1:1 ratio with GV3101 containing TRV1 empty vectors (GV3101/TRV2), in days 1, 7, 14, 21 and 28 post agro-drench respectively; **f**, **g**, **h**, **i** and **j** were agro-drenched with GV3101 vectors containingTRV1 empty (GV3101/TRV1) in days 1,7,14,21 and 28 respectively. **k**, **l**, **m**, **n**, and **o** plants were agro-drenched with GV3101 only, while **p**, **q**, **r**, **s** and **t** were only watered in days 1, 7, 14, 21 and 28 after agro-drench respectively. All scale bars represent 5 cm.

**Figure 2:**
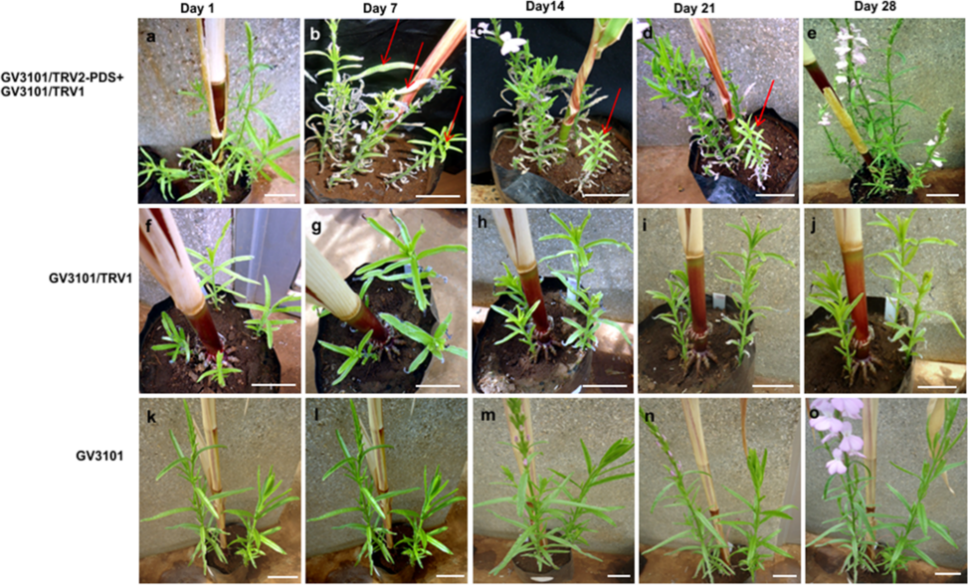
**Viral induced gene silencing (VIGS) via agro-infiltration method on *S. hermonthica*. a**, **b**, **c**, **d**, and **e** are plants agro-infiltrated with *Agrobacterium* strain GV3101 habouring TRV2-PDS (GV3101/TRV2-PDS), and mixed in 1:1 ratio with GV3101 containing TRV1 empty vectors (GV3101/TRV2), in days 1, 7, 14, 21 and 28 after agro-infiltration respectively; **f**, **g**, **h**, **i** and **j** are plants agro-infiltrated with GV3101 vectors containing TRV1 empty (GV3101/TRV1) in days 1, 7, 14, 21 and 28 after infiltration respectively; **k**, **l**, **m**, **n**, and **o** are plants agro-infiltrated with GV3101 only, in days 1, 7, 14, 21 and 28 after agro-infiltration respectively. All scale bars represent 5 cm.

Viral induced technique through agro-drench methods was effective in *S. hermonthica* plants. This was evidenced by down regulation of the PDS gene resulting into photo-bleached phenotypes on the leaves of S*. hermonthica* plants. The bleaching appeared on plants agro-drenched with the *Agrobacterium* strain GV3101 habouring the TRV1 and mixed with GV3101 having TRV2 vector containing the PDS insert (Figure 1a, b, c, d, e). The photo-bleaching effects appeared on the 14th day after agro-drench but the plants recovered on the 28th day post infection (Figure 1c) and (Figure 1e) respectively. There was no photo-bleaching on the control agro-drenched plants leaves (Figure 1f-t). Similarly agro-infiltrated *S. hermonthica* plants developed photo-bleached phenotypes on the leaves of PDS targeted *S. hermonthica* plants. The effects appeared on the 7th day after agro-infiltration (Figure 2b) and the plants recovered on the 28th day post agro-infiltration (Figure 2d). The negative control agro-infiltrated plants did not show photo-bleaching symptoms (Figure 2f-o).

### VIGS induced RNAi is because of down-regulation of *S. hermonthica* PDS

**Figure 3:**
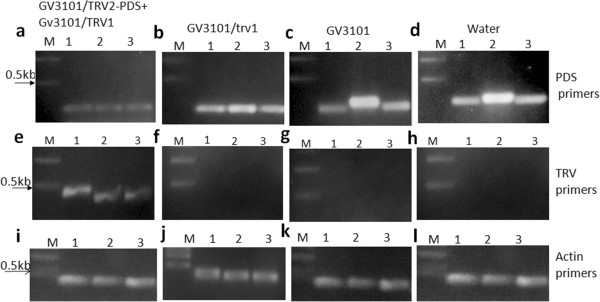
**RT-PCR to confirm silencing of *PDS *gene on *S. hermonthica.*** The PDS and TRV gene specific primers were used. **(a)***S. hermonthica* treated with GV3101 with the TRV2 vector containing the PDS gene insert (GV3101/TRV2-PDS) + GV3101/TRV1) and amplified with PDS primers. Lane (M) represents 1 kb ladder while 1, 2, and 3 are the replicates. **(b)** The cDNA plants treated with GV3101 containing TRV1 empty vectors (GV3101/TRV1) with three replicates (1, 2 and 3) and amplified with PDS primers. **(c)** Three replicates (1, 2, 3) of plants treated with GV3101 empty and amplified with PDS primers. **(d)** Represents plants treated only with water and amplified with PDS primers. **(e)***S. hermonthica* cDNA amplified with TRV primers from plants treated with GV3101/TRV2-PDS + GV3101/TRV1 vectors with three replicates (1, 2, 3). **(f)***S. hermonthica* cDNA from Plants treated with GV3101/TRV1 empty vectors, three replicates (1, 2, and 3) amplified with TRV primers. **(g)** Plants treated with GV3101 empty, three replicates (1, 2, 3) amplified with TRV primers. **(h)** Plants treated with water only, three replicates (1,2,3), cDNA amplified with TRV primers. Images (**i**, **j**, **k** and **l**) are the internal control using Actin primers for the *S. hermonthica* cDNA from plants treated with GV3101/TRV2-PDS + GV3101/TRV1, Plants treated with GV3101/TRV1 only, Plants treated with GV3101 only and plants treated with water only, respectively.

To confirm if silencing of the PDS had occurred in *S. hermonthica* due to infiltration and agro-drench, RT-PCR analysis was done using PDS and TRV gene specific primers. The PDS primers were designed to prime outside the region of homology between the VIGS vector and target mRNA. The RT-PCR of the cDNA from photo-bleached *S. hermonthica* leaves, using PDS primers amplified a 250 bp fragment as expected (Figure 3a). However, fragments were extremely faint due to down regulation of the PDS gene in these plants. The negative control plants treated with GV3101/TRV1, GV3101 empty and those which were only watered had bands of higher intensity indicating the PDS was highly expressed in these plants because there was no down-regulation (Figure 3b, c, d, respectively). Additionally, the cDNA amplification with TRV primers indicated the success of VIGS by amplifying a 400 bp fragment on plants treated with GV3101/TRV2 with the PDS insert (Figure 3e). Amplification of the cDNA from plants treated with; GV3101/TRV1 empty, GV3101 empty and water only did not have the 400 bp fragment with TRV primers (Figure 3f, g, h respectively). The *S. hermonthica* DNA amplification with primers for Actin gene showed the 426 bp fragment indicating the quality of the cDNA of plants from all the four treatments (Figure 3i, j, k, l).

### VIGS efficiency on *S. hermonthica*

Statistical analysis using student’s *t*-test revealed that the results of VIGS were successful on plants targeted for PDS silencing (treated with GV3101/TRV2/PDS + GV3101/TRV1) in *S. hermonthica*. There was indication of photo-bleaching by 60.2 ± 2.9 percentage of *S. hermonthica* plants targeted for PDS plants silencing in Agro-infiltration method, while in agro-drench only 10.3 ± 1.5. None of the negative control treatments indicated photo-bleaching effects in both methods (Table 1).

**Table 1:** **VIGS efficiency in *S. hermonthica***

**Treatment**	**No of transformed plants**	**% of PDS transformed plants (photo-bleached)**	**% of PDS negative plants (not photo-bleached)**
**GV3101/TRV2-PDS + GV3101/TRV1**			
Agro-infiltration	12.9 ± 2.9	60.2 ± 2.9*	39.8 ± 5
Agro-drench	12.2 ± 1.5	10.3 ± 1.5*	89.7 ± 3
**GV3101/TRV1**	8.0 ± 1.5	0.0	100.0*
**GV3101**	8.0 ± 1.0	0.0	100.0*

*Significant values; P ≤ 0.05.

## Discussion

Plants induce homology dependent defense mechanisms in response to attack by virus, therefore engineering a virus into a plant to target a gene of interest results in silencing of the gene through PTGS [29]. The PTGS mechanisms are similar to those of RNA interference in plants [27]. Our experiments demonstrate that VIGS using TRV vectors with the PDS gene resulted in inhibition of carotenoid biosynthesis in *S. hermonthica*. This was evidenced by down-regulation of the *PDS* gene in *S. hermonthica* plants resulting in photo bleaching phenotypes at 7 and 14 days post inoculation in agro-infiltrated and agro-drenched plants respectively. The early appearance of silencing in agro-infiltrated *S. hermonthica* plants as compared to the agro-drenched ones could be ascribed to high efficiency in the delivery of the signals that initiate the innate silencing machinery of PGS in the *S. hermonthica* leaves. In our case the VIGS vector was introduced in *S. hermonthica* via *A. tumefaciens* infection. It is also possible that *A. tumefaciens* was more efficient at transferring the T-DNA containing the viral genome and PDS into the host genome in the cells of the leaves than in the root and stem cells, this could have therefore delayed all the downstream silencing steps in agro-drenched plants [30]. Further these results show that a better mechanism in spreading the silencing signal after introduction exists in *S. hermonthica* leaf tissue than in the root or stem tissue. The translocation of PTGS silencing factor may utilize both short-range cell-to-cell movements through plasmodesmata as well as phloem-associated long-range transport mechanisms [35,36]. The RNA-dependent RNA Polymerase6 (RDR6) is required for long-range transport, possibly by amplifying the silencing signal [33]. From our experiments we are not able to verify if RDR6 in leaf, root and stem cells of *S. hermonthica* is responsible for the difference in the efficiency. The silencing efficiency however has been reported to be proportional to the number of silencing molecules in the cells [37].

*Phytoene desaturase* (PDS) is a key enzyme involved in carotenoid biosynthesis pathway [28]. It’s known that reduced levels of photo-protective carotenoids leads to rapid destruction of chlorophyll by photo-oxidation that results to white or bleached phenotypes [38]. The recovery of the photo-bleached plants at 28 days post inoculation is attributed to the transient nature by which VIGS is expressed in cells. The negative control plants treated with GV3101/TRV1, GV3101 empty and water did not show bleaching characteristic of the PDS silencing effects, because for any silencing to occur, the PDS insert must be contained in TRV2 expression vector that encode the virus coat protein genes responsible for viral replication [39]. The control plants could not therefore initiate the PTGs silencing machinery. The silencing of the PDS was therefore as a result of the infected *S. hermonthica* plants employing the innate PTGS as defense mechanism against the TRV. The PTGS as a response has been widely reported in plants [27,30,34].

This study has established a VIGS protocol that can be used for reverse genetics or functional genomics studies in *S. hermonthica*. This approach also ensures that the gene validation can proceed without laboring with stable transformation of the injurious parasite or its recalcitrant monocot hosts. Although there is limited evidence that genetic material can be exchanged between *S. hermonthica* and its hosts [20,21], the developed tools could be independently used without having to worry about delivery of enough of the silencing molecules through from the host to parasite via the haustoria. In fact once the factors that enable *S. hermonthica* to uncontrollably colonize its host are identified, the parasite could be basted by boasting host defense mechanisms through gene over-expression techniques along the identified pathways. In such a case delivery of resistance molecules will not be in question, as the host will directly exhibit resistance to *S. hermonthica* on attachment*.*

## Conclusion

We have demonstrated that TRV VIGS vectors could be used in functional genomics in the parasitic weed *S. hermonthica.* Although VIGS was more efficient through agro infiltration than agro-drench, using the PDS gene obviously results in an above ground leaf phenotype, it remains to be seen what will be observed during validation in *S. hermonthica* parasitism genes where most of the promising phenotypes are expected to occur in the roots where haustoria colonizes. Finally, with the available genetic resources at the Parasitic Plant Genome Project, and the developed tools will aid in the validation and identification of genes responsible for unabated *S. hermonthica* parasitism. The identification could lead to a variety of transgenic approaches that could lead to development of *S. hermonthica* resistant germplasm not only in maize but in other cereal hosts as well.

## Materials and methods

### VIGS plasmids

**Figure 4:**
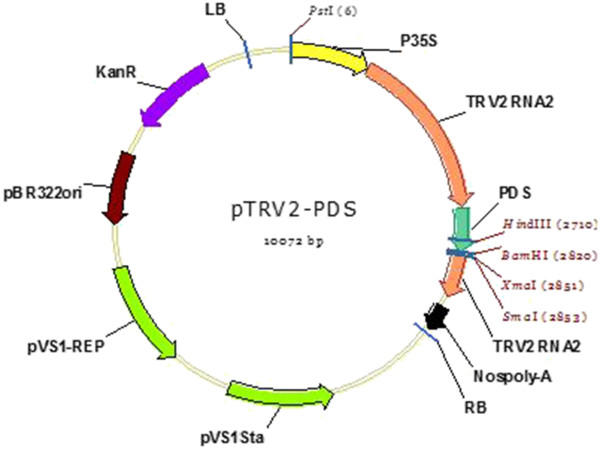
**TRV2 vector with the PDS insert.using the TRV2 vector details provided in the ABRC database **[41]**, the map was constructed using Vector NTI software, version 11.5.2.**

Tobacco rattle virus (TRV)-derived vectors were provided by Prof. Dinesh Kumar from the University of California-Davis. The Tobacco rattle virus contains bipartite positive-sense RNA genome (RNA1 and RNA2). The TRV1 vector represented RNA1 which encodes two viral replication proteins, a movement protein and a seed transmission factor. The provided TRV2 represented the RNA2 and encodes the coat protein and a nematode transmission plant kingdom [40]. The two binary vectors were separately transformed in the *A. tumefaciens* Strain GV3101 which was the delivery vehicle for the viral vectors into plants through agro-inoculation and agro-drench. *Agrobacterium* colonies carrying TRV1, and TRV2 vector with the PDS insert (Figure 4) were grown separately in Luria bertani (LB) liquid media containing Kanamycin 50 mg/l and rifampicin 1 mg/l overnight. From the overnight culture, 1 ml was picked from each tube and again sub-cultured separately for 2 hours in 10 ml LB media containing Kanamycin 50 mg/L and rifampicicn 1 mg/L and 150 μM of acetosyringone to induce the virulence genes. The separate 10 mL liquid cultures were grown at 28°C in darkness until they attained an optical density (OD) of 0.6. The two cultures were then spinned for 15 minutes at 10000 revolutions per minute (RPM) and resuspended in an induction buffer containing 150 μM acetosyringone, 10 mM of MES and 10 mM of MgCl_2,_ adjusted to PH 5.6 and grown again for 2 hours. *Agrobacterium* strains GV3101 containing TRV1 and TRV2-PDS were then mixed in a 1:1 ratio and used in the agro-inoculation experiments (Figure 4).

### Agro-drench

Agro-drench involved applying the mixture of GV3101/TRV2PDS and GV3101/TRV1 (1:1) ratio directly onto the soil adjacent to the crown part of 3–4 week old *S. hermonthica* plants as described by [39] with slight modifications. The experiments involved six plants and were repeated three times. The negative controls were six plants treated with GV3101/TRV1, GV3101, and water separately. All the negative controls were replicated thrice as well. Pictures were taken after every seven days and the numbers of plants showing photo-bleaching effects were recorded.

### Agro-infiltration

For leaf agro-infiltration in *S. hermonthica*, all the young leaves on the upper part of the plant were infiltrated by pricking the lower side of the leaves with a wire brush. Using cotton wool the GV3101/TRV2-PDS and GV3101/TRV1 mixture was gently applied on the pricked leaves until they became fully wet. Eight *S. hermonthica* plants were used and the experiment was repeated three times. The control *S. hermonthica* plants were separately infiltrated with GV3101 empty, GV3101/TRV1 and water only. Plants were photographed every seven days and the number of plants showing photo-bleaching effects recorded.

### Screening for silencing of *phytoene desaturase* gene through reverse transcriptase-polymerase chain reaction (RT-PCR)

Leaf tissues from three of plants from all the treatments were collected and ground in liquid nitrogen using a pestle and mortar. Approximately 20 mg of ground tissues was used for total RNA extraction as per the instructions of the RNeasy® mini kit (Qiagen, Cat no 74104, Valencia. U.S.A). The total RNA was subjected to DNAse treatment and incubated at 37°C for 15 minutes. The total RNA extracted was then reverse transcribed to cDNA synthesis using Superscript™ III first stand synthesis system (Invitrogen, CAT 18080–051, Carlsbad, U.S.A). The first strand cDNA synthesis reactions were primed using random heximers. The synthesized cDNA was then amplified using PDS primers and TRV primers. The PDS primers were (Forward primer 5′-GAGAAACATGGTTCAAAAATGG-3′and reverse primer 5′-AACACAAAAGCATCTCCCTC-3′). The PDS primers were designed to prime outside the region of homology between the VIGS vector and the target mRNA. The TRV primers were (Forward 5′-ACTCACGGGCTAACAGTGCT-3′ and reverse primer 5′-GACGTATCGGACCTCCACTC-3′. The PCR was set with 94°C denaturation temperature, 55°C annealing temperature and 74°C extension temperature for 40 cycles. Gel electrophoresis was performed at 100 volts using 1% of agarose loaded with 5 μl of each sample reaction. Gel pictures were taken under a Ultraviolet light illuminator after a 30 minutes run.

## Abbreviations

SSA: Sub Saharan Africa; PDS: *Phytoene desaturase*; Bp: Base pairs; Kb: Kilobytes; RPM: Revolutions per minute; TRV: Tobacco rattle virus; PTGS: Post transcription gene silencing; OD: Optical density; RNAi: Ribonucleic acid interference; VIGS: Viral induced gene silencing; siRNA: Small interfering ribonucleic acid; T-DNA: Transfer DNA of the tumor-inducing (Ti) plasmid in Agrobacterium tumefaciens; dsRNA: Double stranded ribonucleic acid; ssRNA: Single stranded ribonucleic acid; RDR6: RNA-dependent RNA Polymerase6; LB: Luria bertani; cDNA: Complementary deoxibonucleic acid; nt: Nucleotide.

## Competing interest

The authors declare that they have no competing interests.

## Authors’ contribution

DK performed the experiments and participated in manuscript preparation, SR planned the experiments and participated in manuscript preparation, AA planned the experiments and participated in manuscript preparation. All authors have read and approved the final manuscript.
